# An efficient approach to study multi-polar fuzzy ideals of semirings

**DOI:** 10.1038/s41598-023-49395-5

**Published:** 2024-01-30

**Authors:** Shahida Bashir, Talal Alharbi, Rabia Mazhar, Issra Khalid, Muneeb ul Hassan Afzal, Nauman Riaz Chaudhry

**Affiliations:** 1 Department of Mathematics, University of Gujrat, Gujrat, 50700 Pakistan; 2 Department of Mathematics, College of Science and Arts in Uglat Asugour, Qassim University, Buraydah, Kingdom of Saudi Arabia; 3 Department of Mechanical Engineering, University of Gujrat, Gujrat, 50700 Pakistan; 4 Department of Computer Science, University of Gujrat, Gujrat, 50700 Pakistan

**Keywords:** Engineering, Mathematics and computing

## Abstract

The multi polar fuzzy (m-PF) set has an extensive range of implementations in real world problems related to the multi-polar information, multi-index and multi-attributes data. This paper introduces innovative extensions to algebraic structures. We present the definitions and some important results of *m*-polar fuzzy subsemirings (*m*-PFSSs), *m*-polar fuzzy ideals (*m*-PFIs), *m*-polar fuzzy generalized bi-ideals (*m*-PFGBIs), *m*-polar fuzzy bi-ideals (*m*-PFBIs) and *m*-polar fuzzy quasi-ideals (*m*-PFQIs) in semirings. The main contributions of the paper include the derivation and proof of key theorems that shed light on the algebraic interplay and computational aspects of *m*-polar fuzzy ideals (*m*-PFIs), *m*-polar fuzzy generalized bi-ideals (*m*-PFGBIs), *m*-polar fuzzy bi-ideals (*m*-PFBIs) and *m*-polar fuzzy quasi-ideals (*m*-PFQIs) in semirings along with examples. Moreover, this paper deals with several important properties of *m*-PFIs and characterizes regular and intra-regular semirings by the properties of these ideals.

## Introduction

The theory of fuzzy set is used in many fields including medical diagnosis, artificial intelligence, computer networks and decision making problems^1^. A membership function with a range of $[\mathrm{0,1}]$ is used to illustrate a fuzzy set^2^. Zadeh introduced the concept of fuzzy set in his paper^3^. Rosenfeld applied this theory on groups^4^. Throughout, the history of fuzzy set, there are many types of fuzzy set extensions^5–8^, for example interval-valued fuzzy sets, vague sets etc. To differentiate the irrelevant and contrary elements in a fuzzy set, Zhang^9^ came up with the term BF set on the basis of this consideration. For illustration, profit and loss, effects and side effects of medicines, both are two-sided aspects of a situation. Lee^10^ introduced the idea of bipolar fuzzy ideals. The BF set is in fact an extension of a fuzzy set with a membership degree range $[-\mathrm{1,1}]$. Many researchers have done various works on BF sets^11^.

The BFS and the 2-polar fuzzy set have a natural one-to-one correspondence. Here, the degree of memberships will be positive for each element^12^. The BFS and 2-polar fuzzy set are cryptomorphic concepts, and one of them can be obtained concisely. For more applications, see^13–15^. Let $K=\{\mathrm{1,2},3, \ldots m\}$ is the set of contexts. Then, for each $k\in K$, the satisfaction degree of an element will be signified by an ${\text{m}}$-PF set with respect to *k*th context^16^. For example, the fuzzy set for the property "brilliant" has various interpretations among students of any class.

These sets can also be utilized as a model for clustering or classification and to define multi relation. An ${\text{m}}$-PF sets have lot of usage in decision making, co-operative games, diagnosis datum, etc. We will give an example to demonstrate it.

Let $X=\{v,w,x,y,z\}$ be the set of 5 students. We shall grade them according to six qualities in the form of 6-PF subset given in Table 1.

**Table 1:** Table of qualities with their membership values.

	Talented	Courteous	Hard work	Punctual	Confidence	Responsible
$v$	0.1	0.4	0	0.4	0	0.9
$w$	0.2	0	0.8	0.5	0.5	0.7
$x$	0.5	0.3	0.8	0.6	0.3	0.6
$y$	0.9	0.8	0.4	0.3	0.4	0.8
$z$	0.7	0.6	0.1	0.9	0.7	0.4

As a result, we get a 6-PF subset $\zeta =X\to [\mathrm{0,1}{]}^{6}$ of X such that

$$\zeta (v)=(\mathrm{0.1,0.4,0},\mathrm{0.4,0},0.9)$$

$$\zeta (w)=(\mathrm{0.2,0},\mathrm{0.8,0.5,0.5,0.7})$$

$$\zeta (x)=(\mathrm{0.5,0.3,0.8,0.6,0.3,0.6})$$

$$\zeta (y)=(\mathrm{0.9,0.8,0.4,0.3,0.4,0.8})$$

$$\zeta (z)=(\mathrm{0.7,0.6,0.1,0.9,0.7,0.4}).$$

**Figure 1:**
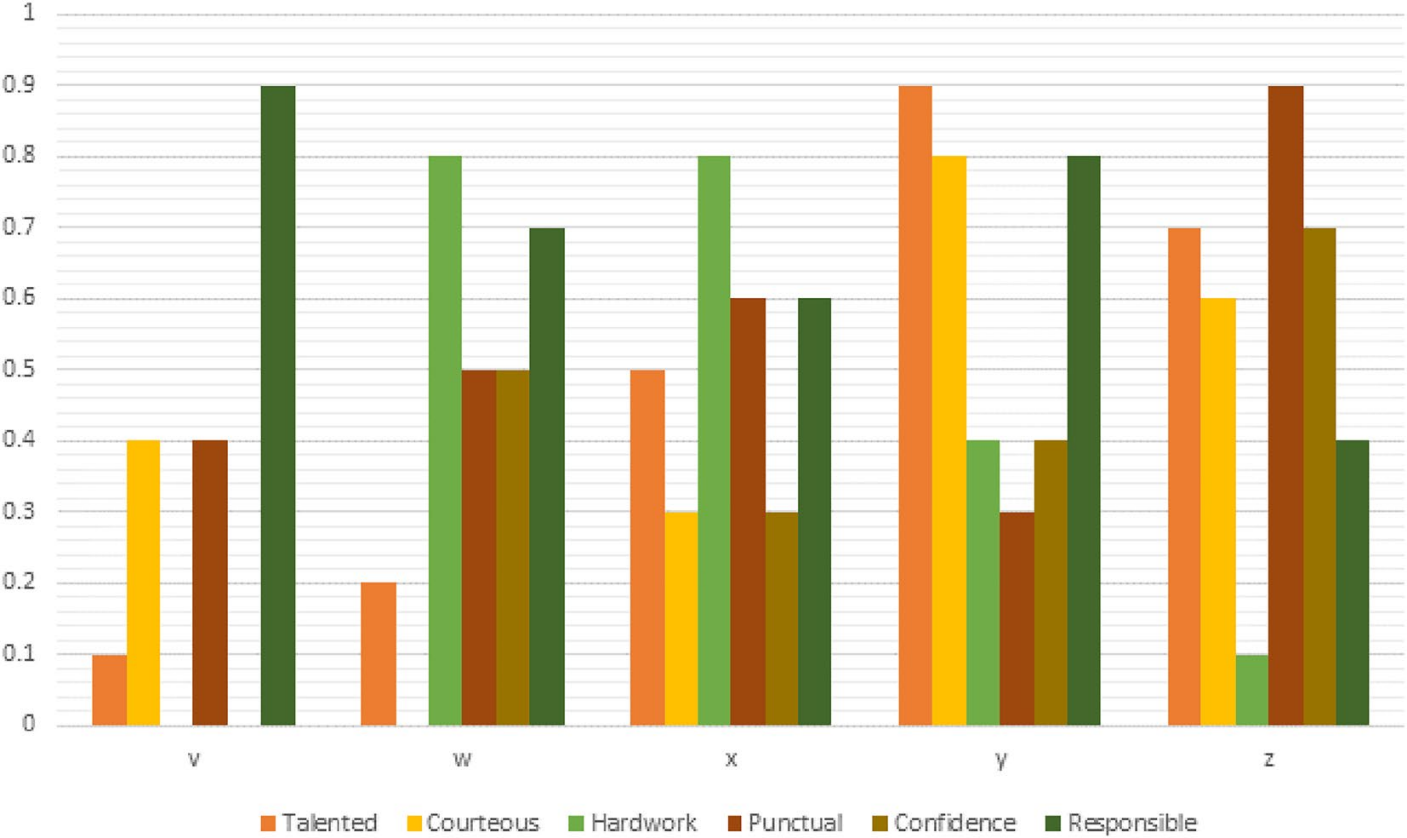
Graphical representation of a 6-PF subset.

The graphical representation of 6-PF subset (shown in Fig. 1):

### Literature review

Now a days, there are working on many useful extensions of fuzzy set in the world^17–21^. The $m$-PF algebraic structures study began with the concept of $m$-PF Lie subalgebras by Akram et al.^22^. The concept of the $m$-PF groups was given by Farooq et al.^23^. Moreover, $m$-PF matroids have been studied in^24^. Further, $m$-PF sets have been studied in different areas, see^25–27^. Al-Masarwah and Ahmad initiated the notions of $m$-PF ideals in^28^ and $m$-polar $(\alpha ,\beta )$-fuzzy ideals in BCK/BCI-algebras^29^. Shabir et al. worked on m-polar fuzzy ideals of LA-semigroup^30^. In 2021, m-polar fuzzy ideals of semigroup are introduced by Bashir et al.^31^. Recently, Bashir et. al have worked on multi-polar fuzzy ideals of ternary semigroup^32^. By extending their work we get a useful approach.

### Organization of the paper

This paper is organized as follows: In Section “Preliminaries”, we give the fundamental definitions related to semiring and fuzzy set on semirings. Section “Characterization of semirings by *m*-polar fuzzy sets” is the main section in which m-PFSS, m-PFI, m-PFBI and m-PFQI of semirings are discussed in detail. In Section “Characterization of regular and intra-regular semirings by *m*-polar fuzzy ideals”, we characterized regular and intra-regular semirings by m-PFIs. In Section “Comparative study” we discuss the comparative study and in last we make conclusion as well as future plans. The list of acronyms is given in Table 2.

**Table 2:** List of acronyms.

Acronyms	Representations
*m*-PF	*m*-Polar fuzzy
BFS	Bipolar fuzzy set
*m*-PFSS	*m*-Polar fuzzy sub semiring
LI (resp. *m*-PFLI)	Left ideal (resp. *m*-Polar fuzzy left ideal)
GBI (resp. *m*-PFGBI)	Generalized bi-ideal (resp. *m*-Polar fuzzy generalized bi-ideal)
BI (resp. *m*-PFBI)	Bi-ideal (resp. *m*-Polarfuzzy Bi-ideal)
QI (resp. *m*-PFQI)	Quasi-ideal (resp. *m*-Polar fuzzy quasi-ideal)
*m*-PFI	*m*-Polar fuzzy ideal

## Preliminaries

This section includes simple but necessary definitions and preliminary results based on semirings that are important in their own right. These are prerequisite for later sections. A non-empty subset $$T$$ of a semring $$(W,+,\cdot )$$ is stated as subsemiring of $$(W,+,\cdot )$$ if $$T$$ itself is a semiring under the same operations. Throughout this research work, $$W$$ will denote semiring unless otherwise specified. In this research work, subsets mean non-empty subsets. A subset $$T$$ of $$W$$ is stated as left ideal $$($$LI) $$({\text{resp}}.$$ right ideal $$($$RI)$$)$$ of $$W$$ if $$T$$ is closed under + and $$WT\subseteq T(TW\subseteq T)$$. If $$T$$ is both LI and RI of $$W$$ then *T* is two-sided ideal or simply an ideal of $$W$$^33^. A subset $$T$$ of $$W$$ is stated as bi-ideal $$($$BI) of $$W$$ if $$T$$ is a subsemiring of $$W$$ and $$TWT\subseteq T$$. A subset $$T$$ of $$W$$ is stated as quasi-ideal $$($$QI) of $$W$$ if $$T$$ is subsemigroup $$(T,+)$$ of $$W$$ and $$WT\cap TW\subseteq T$$^34^. A fuzzy subset $$\zeta$$ of $$W$$ is a function $$\zeta : W\to [\mathrm{0,1}].$$ The BF set has [− 1,1] membership degree range. An $$m$$-PF subset (or $$[\mathrm{0,1}{]}^{m}$$ set) on $$W$$ is mapping $$\zeta : W\to [\mathrm{0,1}{]}^{m}.$$

An $m$-PF set is the $m$-tuple of membership degree function of $W$ that is $\zeta =({\zeta }_{1},{\zeta }_{2},{\zeta }_{3}, \ldots ,{\zeta }_{m})$, where ${\zeta }_{\kappa }$ $: W\to [\mathrm{0,1}]$ is a mapping for all $\kappa \in \{\mathrm{1,2},3, \ldots ,m\}$. An $m$-PF power set of $W$, that is the set of all $m$-PF subsets of $W$ is denoted by $m(W)$. We define relation on $m(W)$ as follows: For any $m$-PF subsets $\zeta =({\zeta }_{1},{\zeta }_{2},{\zeta }_{3},...,{\zeta }_{m})$ and $\xi =({\xi }_{1},{\xi }_{2},{\xi }_{3},...,{\xi }_{m})$ of $W,$ $\zeta \le \xi$ means that ${\zeta }_{\kappa }(l)\le {\xi }_{\kappa }(l)$ for all $l\in W$ and $\kappa \in \{\mathrm{1,2},3,...,m\}.$ The symbols $\zeta \wedge \xi$ and $\zeta \vee \xi$ means $(\zeta \wedge \xi )(l)=\zeta (l)\wedge \xi (l)$ and $(\zeta \vee \xi )(l)=\zeta (l)\vee \xi (l)$ that is $({\zeta }_{\kappa }\wedge {\xi }_{\kappa })(l)={\zeta }_{\kappa }(l)\wedge {\xi }_{\kappa }(l)$ for each $l\in W$ and $\kappa \in \{\mathrm{1,2},3,...,m\}$ and $({\zeta }_{\kappa }\vee {\xi }_{\kappa })(l)={\zeta }_{\kappa }(l)\vee {\xi }_{\kappa }(l)$ for each $l\in W$ and $\kappa \in \{\mathrm{1,2},3,...m\}.$ For any $m$-PF subsets $\zeta =({\zeta }_{1},{\zeta }_{2},{\zeta }_{3},...,{\zeta }_{m})$ and $\xi =({\xi }_{1},{\xi }_{2},{\xi }_{3},...,{\xi }_{m})$ of $W,$ $\zeta \le \xi$ means that ${\zeta }_{\kappa }(l)\le {\xi }_{\kappa }(l)$ for all $l\in W$ and $\kappa \in \left\{\mathrm{1,2},3,...,m\right\}$^1^. Let $\zeta$ be fuzzy subset of $W$ and $t\in (\mathrm{0,1}].$ The set ${\zeta }_{t}=\{l\in W|\zeta (l)\ge t\}$ is stated as level subset of $\zeta$.

Let $\xi$ and $\zeta$ be fuzzy subsets of $W$. Define the fuzzy subset $\xi \circ \zeta$ of $W$ by

$$(\xi \circ \zeta )(l)=\left\{\begin{array}{ll}{\vee }_{l=mn} \{\xi (m)\wedge \zeta (n)\}, & \text{ if }l=mn;\\ 0 & \text{ otherwise}\end{array}\right.$$

for all $$l\in W$$.

Let $\xi$ and $\zeta$ be fuzzy subsets of $W$. Define the fuzzy subset $\xi +\zeta$ of $W$ by

$$\left(\xi +\zeta \right)\left(l)=\right)\left\{\begin{array}{ll}{\vee }_{l=m+n }\{\xi (m)\wedge \zeta (n)\}, & \text{ if }l=m+n;\\ 0 & \text{ otherwise.}\end{array}\right.$$

The next example shows the multiplication and addition of $m$-PF subsets.

### Example 1

Consider a semiring $W=\{u,v,w\}$ under the operations as given in Tables 3 and 4.

**Table 3:** Table of addition.

$+$	$u$	$v$	$w$
$u$	$u$	$v$	$w$
$v$	$v$	$v$	$v$
$w$	$w$	$v$	$w$

**Table 4:** Table of multiplication.

$*$	$u$	$v$	$w$
$u$	$u$	$u$	$u$
$v$	$u$	$u$	$u$
$w$	$u$	$u$	$u$

We define 3-PF subsets $\zeta =({\zeta }_{1},{\zeta }_{2},{\zeta }_{3})$ and $\xi =({\xi }_{1},{\xi }_{2},{\xi }_{3})$ of $W$ as follows: $\zeta (u)=(\mathrm{0.1,0.2,0.1}),\zeta (v)=(\mathrm{0,0},0),\zeta (w)=(\mathrm{0.2,0.3,0.4})$ and $\xi (u)=(\mathrm{0,0},0),\xi (v)=(\mathrm{0,0.1,0.2}),\xi (w)=(\mathrm{0.3,0},0.4).$

By definition, we have

$$({\zeta }_{1}\circ {\xi }_{1})(u)=0.2,({\zeta }_{1}\circ {\xi }_{1})(v)=0,({\zeta }_{1}\circ {\xi }_{1})(w)=0;$$

$$({\zeta }_{2}\circ {\xi }_{2})(u)=0.1,({\zeta }_{2}\circ {\xi }_{2})(v)=0,({\zeta }_{2}\circ {\xi }_{2})(w)=0;$$

$$({\zeta }_{3}\circ {\xi }_{3})(u)=0.4,({\zeta }_{3}\circ {\xi }_{3})(v)=0,({\zeta }_{3}\circ {\xi }_{3})(w)=0.$$

Hence the product of $\zeta =({\zeta }_{1},{\zeta }_{2},{\zeta }_{3})$ and $\xi =({\xi }_{1},{\xi }_{2},{\xi }_{3})$ is defined by

$$(\zeta \circ \xi )(u)=(\mathrm{0.2,0.1,0.4}),(\zeta \circ \xi )(v)=(\mathrm{0,0},0),(\zeta \circ \xi )(w)=(\mathrm{0,0},0).$$

Also,

$$({\zeta }_{1}+{\xi }_{1})(u)=0,({\zeta }_{1}+{\xi }_{1})(v)=0,({\zeta }_{1}+{\xi }_{1})(w)=0.2;$$

$$({\zeta }_{2}+{\xi }_{2})(u)=0,({\zeta }_{2}+{\xi }_{2})(v)=0.1,({\zeta }_{2}+{\xi }_{2})(w)=0;$$

$$({\zeta }_{3}+{\xi }_{3})(u)=0,({\zeta }_{3}+{\xi }_{3})(v)=0.2,({\zeta }_{3}+{\xi }_{3})(w)=0.4.$$

Hence the addition of $\zeta =({\zeta }_{1},{\zeta }_{2},{\zeta }_{3})$ and $\xi =({\xi }_{1},{\xi }_{2},{\xi }_{3})$ is defined by

$$(\zeta +\xi )(u)=(\mathrm{0,0},0),(\zeta +\xi )(v)=(0.,\mathrm{0.1,0.2}),(\zeta +\xi )(w)=(\mathrm{0.2,0},0.4).$$

## Characterization of semirings by ${\varvec{m}}$-polar fuzzy sets

This is our most important section, as here we make most significant contributions. With the help of examples, theorems, and lemmas, the notions of $m$-PFSS, $m$-PFI, $m$-PFGBI, $m$-PFBI, and $m$-PFQI are presented in this phase. An $m$-PF set is wider in structure than BPF set. Throughout the paper, $\delta$ is the $m$-PF subset of $W$ that maps every element of $W$ on $\left(\mathrm{1,1}, \ldots ,1\right)$.

### Definition 1

Let $\zeta =({\zeta }_{1},{\zeta }_{2}, \ldots ,{\zeta }_{m})$ be an *m*-PF subset of $W.$

1. Define ${\zeta }_{t}=\left\{l\in W|\zeta (l)\ge t\right\}$ for all $t$*,*where $t=({t}_{1},{t}_{2}, \ldots ,{t}_{m})\in (\mathrm{0,1}{]}^{m}$*,* then ${\zeta }_{t}$ is called t-cut or a level set. This means ${\zeta }_{\kappa }(l)\ge {t}_{\kappa }$ for all $\kappa \in \{\mathrm{1,2}, \ldots ,m\}$.
2. The support of $\zeta : W\to [\mathrm{0,1}{]}^{m}$ is stated as $Supp( \zeta )=\{l\in W|\zeta (l)>(\mathrm{0,0}, \ldots ,0) m$-tuple} that is ${\zeta }_{\kappa }(l)>0$ for all $\kappa \in \{\mathrm{1,2}, \ldots ,m\}.$

### m-Polar fuzzy subsemirings and ideals in semirings

Here, we define the *m*-PFSS and *m*-PFI of a semirings with example and explain the related lemmas.

#### Definition 2

An $m$-PF subset $\zeta$ of $W$ is stated as $m$-PFSS of $W$ if for all $l,m\in W$ it satisfies the following conditions:

1. $$\zeta (l+m)\ge \zeta (l)\wedge \zeta (m)$$ that is $${\zeta }_{\kappa }(l+m)\ge {\zeta }_{\kappa }(l)\wedge {\zeta }_{\kappa }(m)$$*;*
2. $$\zeta \left(lm\right)\ge \zeta \left(l\right)\wedge \zeta \left(m\right)$$ that is $${\zeta }_{\kappa }(lm)\ge {\zeta }_{\kappa }(l)\wedge {\zeta }_{\kappa }(m)$$

for all $$\kappa \in \{\mathrm{1,2},3,\dots ,m\}.$$

#### Definition 3

An $$m$$-PF subset $$\zeta$$ of $$W$$ is stated as $$m$$-PFLI $$({\text{resp}}. m$$-PFRI$$)$$ of $$W$$ if for all $$l,m\in W$$ it satisfies the following conditions:

1. $$\zeta (l+m)\ge \zeta (l)\wedge \zeta (m)$$ that is $${\zeta }_{\kappa }(l+m)\ge {\zeta }_{\kappa }(l)\wedge {\zeta }_{\kappa }(m)$$*;*
2. $\zeta \left(lm\right)\ge \zeta \left(m\right) (resp. \zeta (lm)\ge \zeta \left(l\right))$ that is ${\zeta }_{\kappa }\left(lm\right)\ge {\zeta }_{\kappa }\left(m\right) \left(resp. {\zeta }_{\kappa }\left(lm\right)\ge {\zeta }_{\kappa }\left(l\right)\right)$ for all $\kappa \in \{\mathrm{1,2},3,\dots ,m\}.$

An $m$-PF subset $\zeta$ of $W$ is called an $m$-PF two-sided ideal or an $m$-PFI of $W$ if it is both $m$-PFLI and $m$-PFRI of $W$. Example 2 is of ideals of 3-PF subset.

#### Example 2

Consider a semiring $W=\{r,s,t\}$ under the operations as given in Tables 5 and 6.

**Table 5:** Table of addition.

+	$r$	$s$	$t$
$r$	$r$	$s$	$t$
$s$	$s$	$s$	$s$
$t$	$t$	$s$	$t$

**Table 6:** Table of multiplication.

$*$	$r$	$s$	$t$
$r$	$r$	$r$	$r$
$s$	$r$	$r$	$r$
$t$	$r$	$r$	$r$

We define 3-PF subset $\zeta =({\zeta }_{1},{\zeta }_{2},{\zeta }_{3})$* of* $W$ as follows:

$$\zeta (r)=(\mathrm{0.2,0},\mathrm{3,0.2}),\zeta (s)=(\mathrm{0,0},0)$$ and $$\zeta (t)=(\mathrm{0.1,0.2,0.1}).$$

Clearly, $\zeta =({\zeta }_{1},{\zeta }_{2},{\zeta }_{3})$ is both a 3-PFLI and 3-PFRI of ${\text{W}}$. Hence, $\upzeta$ is 3-PF two-sided ideal.

#### Definition 4

Let $T\subseteq W.$ Then the $m$-polar characteristic function ${C}_{T} : W\to [\mathrm{0,1}{]}^{m}$ of $T$ is defined as

$${C}_{T}\left(l\right)=\left\{\begin{array}{c}\left(\mathrm{1,1},\dots ,1\right),m\text{-tuple if }l\in T;\\ \left(\mathrm{0,0},\dots ,0\right),m\text{-tuple if }l\notin T.\end{array}\right.$$

#### Lemma 1

*Let *$T$* and *$U$* be subsets of *$W$*. Then the followings hold*^31^.

1. $${C}_{T}\vee {C}_{U}={C}_{T\cup U};$$
2. $${C}_{T}\wedge {C}_{U}={C}_{T\cap U};$$
3. $${C}_{T}\circ {C}_{U}={C}_{TU}.$$

#### Lemma 2

*Let* $T$ *be a subset of* $W.$ *Then the following assertions are true.*

1. $T$ *is subsemiring of* $W$ *if and only if* ${C}_{T}$ *is* $m$ *-PFSS of* $W.$
2. $$T$$ *is LI* $$($$ *resp. RI, two-sided ideal* $$)$$ *of* $$W$$ *if and only if* $${C}_{T}$$ *is an* $$m$$ *-PFLI* $$($$ *resp.* $$m$$ *-PFRI,* $$m$$ *-PF two-sided ideal* $$)$$ *of* $$W.$$

#### Proof

(1) Let $T$ is subsemiring of $W$. We have to show that ${C}_{T}(lm)\ge {C}_{T}(l)\wedge {C}_{T}(m)$ and ${C}_{T}(l+m)\ge {C}_{T}(l)\wedge {C}_{T}(m)$ for all $l,m\in W$.

Case 1: Let $l,m\in T.$ This implies that ${C}_{T}(l)={C}_{T}(m)=(\mathrm{1,1}, \ldots ,1)$. Since $T$ is subsemiring of $W$. Then $lm\in T$ and $l+m\in T,$ this implies that ${C}_{T}(lm)=(\mathrm{1,1}, \ldots ,1)$ and ${C}_{T}\left(l+m\right)=\left(\mathrm{1,1}, \ldots ,1\right).$ So, ${C}_{T}\left(lm\right)={C}_{T}(l)\wedge {C}_{T}(m)$ and ${C}_{T}\left(l+m\right)={C}_{T}(l)\wedge {C}_{T}(m).$

Case 2: Let $l,m\notin T$. This implies that ${C}_{T}(l)={C}_{T}(m)=(\mathrm{0,0}, \ldots ,0).$ As $l,m\notin T$ and $T$ is subsemiring of $W$. So, ${C}_{T}(lm)\ge {C}_{T}(l)\wedge {C}_{T}(m)$ and ${C}_{T}(l+m)\ge {C}_{T}(l)\wedge {C}_{T}(m).$

Case 3: Let $l\in T$ and $m\notin T.$ This implies that ${C}_{T}(l)=(\mathrm{1,1}, \ldots ,1)$ and ${C}_{T}(m)=(\mathrm{0,0}, \ldots ,0).$ Clearly, ${C}_{T}(lm)\ge (\mathrm{0,0}, \ldots ,0)={C}_{T}(l)\wedge {C}_{T}(m)$ and ${C}_{T}(l+m)\ge (\mathrm{0,0}, \ldots ,0)={C}_{T}(l)\wedge {C}_{T}(m)$.

Conversely, suppose ${C}_{T}$ is $m$-PFSS of $W.$ Let $l,m\in T.$ Then ${C}_{T}(l)=(\mathrm{1,1}, \ldots ,1)$ and ${C}_{T}(m)=(\mathrm{1,1}, \ldots ,1)$.

By definition ${C}_{T}(lm)\ge {C}_{T}(l)\wedge {C}_{T}(m)=(\mathrm{1,1}, \ldots ,1)\wedge (\mathrm{1,1}, \ldots ,1)=(\mathrm{1,1}, \ldots ,1),$ we have ${C}_{T}(lm)=(\mathrm{1,1}, \ldots ,1).$ Also by definition ${C}_{T}(l+m)\ge {C}_{T}(l)\wedge {C}_{T}(m)=(\mathrm{1,1}, \ldots ,1)\wedge (\mathrm{1,1}, \ldots ,1)=(\mathrm{1,1}, \ldots ,1),$ we have ${C}_{T}(l+m)=(\mathrm{1,1}, \ldots ,1).$ This implies that $lm\in T$ and $l+m\in T$ that is $T$ is a subsemiring of $W.$

(2) Suppose that $T$ is an LI of $W$. We have to show that ${C}_{T}(l+m)\ge {C}_{T}(l)\wedge {C}_{T}(m)$ and ${C}_{T}(lm)\ge {C}_{T}(m)$ for all $l,m\in W.$ We consider the following two cases.

Case 1: Let $m\in T$ and $l\in W$. Then ${C}_{T}(m)=(\mathrm{1,1}, \ldots ,1)$. Since $T$ is an LI of $W$, so $lm\in T$ implies that ${C}_{T}(lm)=(\mathrm{1,1}, \ldots ,1)$. Hence ${C}_{T}(lm)\ge {C}_{T}(m).$

Case 2: Let $m\notin T$ and $l\in W.$ Then ${C}_{T}(m)=(\mathrm{0,0}, \ldots ,0)$. Clearly ${C}_{T}(lm)\ge {C}_{T}(m).$

Conversely, let ${C}_{T}$ is an $m$-PFLI of $W$. Let $l\in W$ and $m\in T.$ Then ${C}_{T}(m)=(\mathrm{1,1}, \ldots ,1).$ By definition, ${C}_{T}(lm)\ge {C}_{T}(m)=(\mathrm{1,1}, \ldots ,1).$ We have ${C}_{T}(lm)=(\mathrm{1,1}, \ldots ,1)$. This implies that $lm\in T$, that is $T$ is LI of $W.$

Similarly, we can prove for RI and two sided ideal.□

#### Lemma 3

*Let* $\zeta =({\zeta }_{1},{\zeta }_{2}, \ldots ,{\zeta }_{m})$ *be an* $m$ *-PF subset of* $W$ *. Then the following assertions are true.*

1. $\zeta$* is an m-PFSS of *$W$* if and only if* $\zeta \circ \zeta \le \zeta$ *and* $\zeta +\zeta \le \zeta ;$
2. $\zeta$ *is an m-PFLI of *$W$* if and only if* $\delta \circ \zeta \le \zeta$ *and* $\zeta +\zeta \le \zeta ;$
3. $\zeta$ *is an m-PFRI of *$W$* if and only if* $\zeta \circ \delta \le \zeta$ *and* $\zeta +\zeta \le \zeta ;$
4. $\zeta$ *is an m-PF two-sided ideal of *$W$* if and only* $\delta \circ \zeta \le \zeta , \zeta \circ \delta \le \zeta$ *and* $\zeta +\zeta \le \zeta .$

#### Proof

(1) Let $\zeta =({\zeta }_{1},{\zeta }_{2}, \ldots ,{\zeta }_{m})$ is an $m$-PFSS of $W$ that is $({\zeta }_{\kappa }(l+m)\ge \{{\zeta }_{\kappa }(l)\wedge {\zeta }_{\kappa }(m)\}$ and ${\zeta }_{\kappa }(lm)\ge {\zeta }_{\kappa }(l)\wedge {\zeta }_{\kappa }(m)$ for all $l,m\in W$ and $\kappa \in \{\mathrm{1,2}, \ldots ,m\}.$ Let $a\in W$, if $a$ is not expressible as $a=b+c$ for some $b,c\in W$. Then $(\zeta +\zeta )(a)=0.$ Hence $(\zeta +\zeta )(a)\le \zeta (a).$ But if $a$ is expressible as $a=l+m$ for some $l,m\in W$. Then

$$\begin{aligned} ({\zeta }_{\kappa }+{\zeta }_{\kappa })(a)&={\vee }_{a=l+m}\{{\zeta }_{\kappa }\left(l\right)\wedge {\zeta }_{\kappa }\left(m\right)\}\\ & \le {\vee }_{a=l+m}\left\{{\zeta }_{\kappa }\left(l+m\right)\right\} \\ & ={\zeta }_{\kappa }(a)\text{ for all }\kappa \in \{\mathrm{1,2}, \ldots ,m\} \end{aligned}$$

Hence, $\zeta +\zeta \le \zeta .$ Also, let $a\in W.$ If $a$ is not expressible as $a= bc$ for some $b,c\in W.$ Then $(\zeta \circ \zeta )(a)=0.$ Hence $(\zeta \circ \zeta )(l)\le \zeta (l).$ But if $a$ is expressible as $a=lm$ for some $l,m\in W.$ Then

$$\left({\zeta }_{\kappa }\circ {\zeta }_{\kappa }\right)\left(a\right)={\vee }_{a=lm}\left\{{\zeta }_{\kappa }\left(l\right)\wedge {\zeta }_{\kappa }\left(m\right)\right\}\le {\vee }_{a=lm}\{{\zeta }_{\kappa }(lm)\}={\zeta }_{\kappa }(a)\text{ for all }\kappa \in \{\mathrm{1,2}, \ldots ,m\}.$$

Hence, $$\zeta \circ \zeta \le \zeta .$$

Conversely, let $\zeta \circ \zeta \le \zeta$ and $\zeta +\zeta \le \zeta .$ We show that $\zeta$ is $m$-PFSS of $W$. Let $l,m\in W.$ Then ${\zeta }_{\kappa }\left(l+m\right)\ge \left({\zeta }_{\kappa }+{\zeta }_{\kappa }\right)\left(l+m\right)={\vee }_{l+m=e+f}\{{\zeta }_{\kappa }(e)\wedge {\zeta }_{\kappa }(f))\}\ge \{{\zeta }_{\kappa }(l)\wedge {\zeta }_{\kappa }(m)\}\text{ for all }\kappa \in \{\mathrm{1,2}, \ldots ,m\}.$

So, $${\zeta }_{\kappa }(l+m)\ge {\zeta }_{\kappa }(l)\wedge {\zeta }_{\kappa }(m)$$. Also, $${\zeta }_{\kappa }(lm)\ge ({\zeta }_{\kappa }\circ {\zeta }_{\kappa })(lm)={\vee }_{lm=ef}\{{\zeta }_{\kappa }(e)\wedge {\zeta }_{\kappa }(f)\}\ge {\zeta }_{\kappa }(l)\wedge {\zeta }_{\kappa }(m) \text{for all }\kappa \in \{\mathrm{1,2}, \ldots ,m\}.$$

So, ${\zeta }_{\kappa }(lm)\ge {\zeta }_{\kappa }(l)\wedge {\zeta }_{\kappa }(m).$ Thus $\zeta$ is an $m$-PFSS of $W.$

Similarly, we can prove the parts $\left(2\right), \left(3\right) {\text{and}} (4).$□

#### Lemma 4

*The following assertions are true in W.*

1. *Let* $\zeta =({\zeta }_{1},{\zeta }_{2}, \ldots ,{\zeta }_{m})$ *and* $\xi =({\xi }_{1},{\xi }_{2}, \ldots ,{\xi }_{m})$ *be* $m$ *-PFSS of* $W$ *. Then* $\zeta \wedge \xi$ *is also an* $m$ *-PFSS of* $W;$
2. *Let* $\zeta =({\zeta }_{1},{\zeta }_{2}, \ldots ,{\zeta }_{m})$ *and* $\xi =({\xi }_{1},{\xi }_{2}, \ldots ,{\xi }_{m})$ *be two* $m$ *-PFLI* $($ *resp.* $m$ *-PFRI,* $m$ *-PF two-sided ideal) of* $W$ *. Then* $\zeta \wedge \xi$ *is also an* $m$ *-PFLI* $($ *resp.* $m$ *-PFRI,* $m$ *-PF two-sided ideal) of* $W.$

#### Proof

Straightforward.□

#### Preposition 1

*Let *$$\zeta =({\zeta }_{1},{\zeta }_{2}, \ldots ,{\zeta }_{m})$$* be an m-PF subset of *$$W$$*. Then *$$\zeta$$* is an m-PFSS *$$($$*resp.*$$m$$*-PFLI, *$$m$$*-PFRI, *$$m$$*-PF two-sided ideal*$$)$$* of *$$W$$* if and only if *$${\zeta }_{t}=\left\{l\in W|\zeta (l)\ge t\right\}\ne \varphi$$* is a subsemiring *$$($$*resp. LI, RI, two-sided ideal*$$)$$* of *$$W$$* for all *$$t=({t}_{1},{t}_{2}, \ldots ,{t}_{m})\in (\mathrm{0,1}{]}^{m}$$.

#### Proof

Let $\zeta$ be an $m$-PFSS of $W$ and let $l,m\in {\zeta }_{t}.$ Then ${\zeta }_{\kappa }(l)\ge {t}_{\kappa }$ and ${\zeta }_{\kappa }(m)\ge {t}_{\kappa }$ for all $\kappa \in (\mathrm{1,2}, \ldots ,m).$ Since $\zeta$ is an $m$-PFSS of $W.$ We have ${\zeta }_{\kappa }(l+m)\ge {\zeta }_{\kappa }(l)\wedge {\zeta }_{\kappa }(m)\ge {t}_{\kappa }\wedge {t}_{\kappa }\ge {t}_{\kappa }$ and ${\zeta }_{\kappa }(lm)\ge {\zeta }_{\kappa }(l)\wedge {\zeta }_{\kappa }(m)\ge {t}_{\kappa }\wedge {t}_{\kappa }\ge {t}_{\kappa }$ for all $\kappa \in (\mathrm{1,2}, \ldots ,m).$ Thus $l+m\in {\zeta }_{t} \mathrm{and }lm\in {\zeta }_{t}.$ Hence, ${\zeta }_{t}$ is a subsemiring of $W$.

Conversely, let ${\zeta }_{t}$ is a subsemiring of $W$. For $l,m\in W$. Suppose on contrary, that ${\zeta }_{t}$ is not $m$-PFSS of $W,$ such that ${\zeta }_{\kappa }(l+m)<{\zeta }_{\kappa }(l)\wedge {\zeta }_{\kappa }(m)$ and take ${t}_{\kappa }\in [\mathrm{0,1}{]}^{m}$ such that ${t}_{\kappa }={\zeta }_{\kappa }(l)\wedge {\zeta }_{\kappa }(m).$ Then $l,m\in {\zeta }_{t}$ but $l+m\notin {\zeta }_{t}.$

Hence ${\zeta }_{\kappa }(l+m)\ge {\zeta }_{\kappa }(l)\wedge {\zeta }_{\kappa }(m).$ Also, for ${\zeta }_{\kappa }(lm)\ge {\zeta }_{\kappa }(l)\wedge {\zeta }_{\kappa }(m)$ suppose on contrary ${\zeta }_{\kappa }(lm)<{\zeta }_{\kappa }(l)\wedge {\zeta }_{\kappa }(m)$ and take ${t}_{\kappa }\in [\mathrm{0,1}{]}^{m}$ such that ${t}_{\kappa }={\zeta }_{\kappa }(l)\wedge {\zeta }_{\kappa }(m).$ Then $l,m\in {\zeta }_{t}$ but $lm\notin {\zeta }_{t}.$ So ${\zeta }_{\kappa }(lm)\ge {\zeta }_{\kappa }(l)\wedge {\zeta }_{\kappa }(m).$ Hence $\zeta$ be a $m$-PFSS of $W.$

Other cases can be proved on the same lines.

Hence, we have proved that the intersection of any two *m*-PFSSs (resp. *m*-PFIs) is also an *m*-PFSS (resp. *m*-PFI). Also, if an *m*-PF subset of $W$ is *m*-PFSS (resp. *m*-PFIs) then its level set is subsemiring (resp. ideal) and vice versa.□

### *m-*Polar fuzzy generalized bi-ideals in semirings

Now, we define the *m*-PFGBI of semirings.

#### Definition 5

An $m$-PF subset $\zeta$ of $W$ is stated as $m$-PFGBI of $W.$ If for all $l,m,n\in W$ it satisfies the following condition: $\zeta (lmn)\ge \zeta (l)\wedge \zeta (n)$ that is ${\zeta }_{\kappa }(lmn)\ge {\zeta }_{\kappa }(l)\wedge {\zeta }_{\kappa }(n)$* for all* $\kappa \in \{\mathrm{1,2},3,\dots ,m\}.$

#### Lemma 5

*A subset* $T$ *of* $W$ *is a GBI of* $W$ *if and only if* ${C}_{T}$ *is an m-PFGBI of* $W.$

#### Proof

Similar to Lemma 2.□

#### Lemma 6

*An* $m$ *-PFSS* $\zeta$ *of* $W$ *is an* $m$ *-PFGBI of* $W$ *if and only if* $\zeta \circ \delta \circ \zeta \le \zeta .$

#### Proof

Let $\zeta =({\zeta }_{1},{\zeta }_{2},...,{\zeta }_{W})$ be an $m$-PFGBI of $W$. Let $n\in W.$ If ${\text{n}}$ is not expressible as $n=ab$ for some $a,b\in W,$ then $\zeta \circ \delta \circ \zeta \le \zeta$. But if $n$ is expressible as $n=lm$ for some $l,m\in W.$ Then for all $\kappa \in \{\mathrm{1,2},...,m\}$ we have.

$$\begin{aligned} ({\zeta }_{\kappa }\circ {\delta }_{\kappa }\circ {\zeta }_{\kappa })(n) & ={\vee }_{n=lm}\{({\zeta }_{\kappa }\circ {\delta }_{\kappa })(l)\wedge {\zeta }_{\kappa }(m)\} \\ & ={\vee }_{n=lm}\{\{{\vee }_{l=uv}\left\{\left({\zeta }_{\kappa }\left(u\right)\wedge {\delta }_{\kappa }\left(v\right)\right\}\wedge {\zeta }_{\kappa }\left(m\right)\right\} \\ & ={\vee }_{n=lm}\{{\vee }_{l=uv}({\zeta }_{\kappa }(u)\wedge {\zeta }_{\kappa }(m)\} \\ & \le {\vee }_{n=lm}\{{\vee }_{l=uv}({\zeta }_{\kappa }(uvm)\} \\ & ={\vee }_{n=lm}{\zeta }_{\kappa }(lm) \\ & ={\zeta }_{\kappa }\left(n\right) \, \text{for all} \, \kappa \in \{\mathrm{1,2}, \ldots ,m\}. \end{aligned}$$

Hence $(\zeta \circ \delta \circ \zeta )\le \zeta$. Conversely, let $\zeta$ satisfies $\zeta \circ \delta \circ \zeta \le \zeta$ and $l,m,n\in W.$ Now,

$$\begin{aligned} {\zeta }_{k}(lmn) & \ge ({\zeta }_{k}\circ {\delta }_{k}\circ {\zeta }_{k})(lmn) \\ & ={\vee }_{lmn=xy}\{{(\zeta }_{k}\circ {\delta }_{k})(x)\wedge {\zeta }_{k}(y)\} \\ & \ge \{{(\zeta }_{k}\circ {\delta }_{k})(lm)\wedge {\zeta }_{k}(n)\} \\ & ={\vee }_{lm=ef}\{({\zeta }_{k})(e)\wedge {\delta }_{k}(f)\}\wedge {\zeta }_{k}(n) \\ & \ge \{({\zeta }_{k})(l)\wedge {\delta }_{k}(m)\}\wedge {\zeta }_{k}(n) \\ & =\left\{{\zeta }_{k}\left(l\right)\wedge {\zeta }_{k}\left(n\right)\right\} \, \text{for all} \, k\in \{\mathrm{1,2}, \ldots ,m\}. \end{aligned}$$

Hence, $$\zeta (lmn)\ge {\zeta }_{k}(l)\wedge {\zeta }_{k}(n)$$.□

#### Proposition 2

*Let* $\zeta =({\zeta }_{1},{\zeta }_{2}, \ldots ,{\zeta }_{m})$ *be an* $m$ *-PFSS of* $W$ *. Then* $\zeta$ *is an* $m$ *-PFGBI of* $W$ *if and only if* ${\zeta }_{t}=\left\{l\in W|\zeta (l)\ge t\right\}\ne \varphi$ *is a GBI of* $W$ *for all* $t=({t}_{1},{t}_{2}, \ldots ,{t}_{m})\in (\mathrm{0,1}{]}^{m}$ *.*

#### Proof

Let $\zeta =({\zeta }_{1},{\zeta }_{2},...,{\zeta }_{m})$ be an $m$-PFSS of $W$ and $\zeta$ is an $m$-PFGBI of $W$. Now let $l,n\in {\zeta }_{t}$ and $m\in W$ .Then ${\zeta }_{\kappa }(l)\ge {t}_{k}$ and ${\zeta }_{\kappa }(n)\ge {t}_{k}$ for all $\in (\mathrm{1,2},...,m)$ . Since $\zeta$ is $m$-PFGBI we have ${\zeta }_{\kappa }(lmn)\ge {\zeta }_{\kappa }(l)\wedge {\zeta }_{\kappa }(n)\ge {t}_{k}\wedge {t}_{k}={t}_{k}$ for all $\kappa \in (\mathrm{1,2}, \ldots ,m).$ Thus we have $lmn\in {\zeta }_{t}.$ So ${\zeta }_{t}$ is GBI of $W$.

Conversely, let ${\zeta }_{t}$ is a GBI of $W.$ On contrary let $\zeta$ is not $m$-PFGBI of $W$. Suppose $l,m,n\in W$ such that ${\zeta }_{\kappa }(lmn)<{\zeta }_{\kappa }(l)\wedge {\zeta }_{\kappa }(n)$ for some $\kappa \in (\mathrm{1,2},...,m).$ Take ${t}_{k}={\zeta }_{\kappa }(l)\wedge {\zeta }_{\kappa }(n).$ Then $l,n\in W$ but $lmn\notin {\zeta }_{t}.$ Which is contradiction. so, ${\zeta }_{\kappa }(lmn)\ge {\zeta }_{\kappa }(l)\wedge {\zeta }_{\kappa }(n)$. Hence, $\zeta$ is an $m$-PFGBI of $W.$

Here, it is shown that if an *m*-PF subset of $W$ is *m*-PFGBI then its level set is GBI and vice versa.□

### *m-*Polar fuzzy bi-ideals in semirings

Here, we define *m-*PFBIs in semirings.

#### Definition 6

An $m$-PF subset $\zeta$ of $W$ is stated as $m$-PFBI of $W,$ if for all $l,m,n\in W$ it satisfies the following conditions: $\zeta (l+m)\ge \zeta (l)\wedge \zeta (m)$*, *$\zeta (lm)\ge \zeta (l)\wedge \zeta (m)$* and* $\zeta (lmn)\ge \zeta (l)\wedge \zeta (n)$ that is ${\zeta }_{\kappa }(l+m)\ge {\zeta }_{\kappa }(l)\wedge {\zeta }_{\kappa }(m)$*, *${\zeta }_{\kappa }\left(lm\right)\ge {\zeta }_{\kappa }\left(l\right)\wedge {\zeta }_{\kappa }\left(m\right)$* and *${\zeta }_{\kappa }(lmn)\ge {\zeta }_{\kappa }(l)\wedge {\zeta }_{\kappa }(n)$* for all* $\kappa \in \{\mathrm{1,2},3,\ldots ,m\}.$

#### Lemma 7

*A subset* $T$ *of* $W$ *is a BI of* $W$ *if and only if* ${C}_{T}$ *is an m-PFBI of* $W.$

#### Proof

Similar to Lemmas 2 and 5.□

#### Lemma 8

*An* $m$ *-PFSS* $\zeta$ *of* $W$ *is an* $m$ *-PFBI of* $W$ *if and only if*

1. $$\zeta +\zeta \le \zeta ;$$
2. $$\zeta \circ \zeta \le \zeta ;$$
3. $$\zeta \circ \delta \circ \zeta \le \zeta .$$

#### Proof

The proofs of (1) and (2) are follows from the proof of Lemma 3, and proof of (3) follows from Lemma 6.□

#### Proposition 3

*Let* $\zeta =({\zeta }_{1},{\zeta }_{2}, \ldots ,{\zeta }_{m})$ *be an* $m$ *-PFSS of* $W$ *. Then* $\zeta$ *is an* $m$ *-PFBI of* $W$ *if and only if* ${\zeta }_{t}=\left\{l\in W|\zeta (l)\ge t\right\}\ne \varphi$ *is a BI of* $W$ *for all* $t=({t}_{1},{t}_{2}, \ldots ,{t}_{m})\in (\mathrm{0,1}{]}^{m}$ *.*

#### Proof

Follows from Proposition 2.□

#### Remark 1

Every m-PFBI of $W$ is an m-PFGBI of $W$

### *m*-Polar fuzzy quasi-ideal in semirings

Now, we define m-PFQI of semirings.

#### Definition 7

An *m*-PF subset $\zeta$ *of* $W$ is called *m*-PFQI *of* $W$ if for all $l,m\in W$*,* $\zeta \left(l+m\right)\ge \zeta \left(l\right)\wedge \zeta \left(m\right)$ *and* $\zeta \circ \delta \wedge \delta \circ \zeta \le \zeta$ that is ${\zeta }_{\kappa }\left(l+m\right)\ge {\zeta }_{\kappa }\left(l\right)\wedge {\zeta }_{\kappa }\left(m\right)$ and ${\zeta }_{\kappa }\circ {\delta }_{\kappa }\wedge {\delta }_{\kappa }\circ {\zeta }_{\kappa }\le {\zeta }_{\kappa }$ for all $\kappa \in \{\mathrm{1,2},3,\dots ,m\}.$

#### Lemma 9

*Let* $T$ *be a subset of* $W$ *. Then* $T$ *is QI of* $W$ *if and only if* ${C}_{T}$ *is an m-PFQI of* $W.$

#### Proof

Let $T$ be a QI of $W$, we have to show that (i) ${C}_{T}(l+m)\ge {C}_{T}(l)\wedge {C}_{T}(m)$ and (ii) $(({C}_{T}\circ \delta )\wedge (\delta \circ {C}_{T}))(l)\le {C}_{T}(l)$ for all $l,m\in W.$

For the parts $(i)$ see Lemma 2.

$$ii)$$ For $$l\in W,$$ consider the following two cases.

Case 1: If $l\in T$ we have ${C}_{T}(l)=(\mathrm{1,1}, \ldots ,1)\ge ({C}_{T}\circ \delta )\wedge (\delta \circ {C}_{T})(l).$ Hence $({C}_{T}\circ \delta )\wedge (\delta \circ {C}_{T})(l)\le {C}_{T}(l).$

Case 2: If $l\notin T$ then $l\notin TW\cap WT.$ This implies that $l\ne ab$ or $l\ne cd$ for all $a\in T,b\in W,c\in W,d\in T.$ Thus either $({C}_{T}\circ \delta )(l)=(\mathrm{0,0}, \ldots ,0)$ or $(\delta \circ {C}_{T})(l)=(\mathrm{0,0}, \ldots ,0),$ that is $\left(\left({C}_{T}\circ \delta \right)\wedge \left(\delta \circ {C}_{T}\right)\right)\left(l\right)=0\le {C}_{T}(l).$

Hence $$({C}_{T}\circ \delta )\wedge (\delta \circ {C}_{T})\le {C}_{T}$$.

Conversely, suppose ${C}_{T}$ is an m-PFQI of ${\text{W}}$ and we have to prove that $T$ is a QI of $W.$ Let $l,m\in T.$ Then ${C}_{T}(l)=(\mathrm{1,1},...,1)$ and ${C}_{T}(m)=(\mathrm{1,1},...,1)$.

By definition ${C}_{T}(l+m)\ge {C}_{T}(l)\wedge {C}_{T}(m)=(\mathrm{1,1}, \ldots ,1)\wedge (\mathrm{1,1}, \ldots ,1)=(\mathrm{1,1}, \ldots ,1),$ we have ${C}_{T}(l+m)=(\mathrm{1,1}, \ldots ,1).$ This implies that $l+m\in T.$ Again, let $n\in TW\cap WT.$ Then $n=el$ and $n=fm$ where $l,m\in W$ and $e,f\in T.$

Since ${C}_{T}$ is *m*-PFQI of $W$. So,

$$\begin{aligned} {C}_{T}\left(n\right) & \ge \left(\left({C}_{T}\circ \delta \right)\wedge \left(\delta \circ {C}_{T}\right)\right)\left(n\right) \\ & =\left({C}_{T}\circ \delta \right)\left(n\right)\wedge \left(\delta \circ {C}_{T}\right)\left(n\right) \\ & ={\vee }_{n=uv}\{{C}_{T}(u)\wedge \delta (v)\}\wedge \{{\vee }_{n=pq}\{\delta (p)\wedge {C}_{T}(q)\}\} \end{aligned}$$

$$\text{since }n=el\text{ and }n=fm\ge \left\{{C}_{T}\left(e\right)\wedge \delta \left(l\right)\right\}\wedge \left\{\delta \left(f\right)\wedge {C}_{T}\left(m\right)\right\}=(\mathrm{1,1}, \ldots ,1).$$

Thus $${C}_{T}(n)=(\mathrm{1,1}, \ldots ,1).$$ Hence $$n\in T.$$

So, $T$ is QI of $W.$□

#### Remark 2

Every m-PFQI of $W$ is an m-PFSS of $W$.

#### Proof

Let $\zeta =({\zeta }_{1},{\zeta }_{2}, \ldots ,{\zeta }_{m})$ be an *m*-PFQI of $W.$ To prove $\zeta$ is *an m-PFSS of* $W,$ it is enough to satisfy the condition, $\zeta \left(lm\right)\ge \zeta \left(l\right)\wedge \zeta \left(m\right)$ for all $l,m\in W.$

Since $\zeta$ is *m*-PFQI so for $\kappa \in \left\{\mathrm{1,2}, \ldots ,m\right\},$

$$\begin{aligned} {\zeta }_{\kappa }(lm) & \ge (({\zeta }_{\kappa }\circ {\delta }_{\kappa })\wedge ({\delta }_{\kappa }\circ {\zeta }_{\kappa }))(lm) \\ & =({\zeta }_{\kappa }\circ {\delta }_{\kappa })(lm)\wedge ({\delta }_{\kappa }\circ {\zeta }_{\kappa })(lm) \\ & =\{{\vee }_{lm=uv}\{{\zeta }_{\kappa }(u)\wedge {\delta }_{\kappa }(v)\}\}\wedge \{{\vee }_{lm=pq}\{{\delta }_{\kappa }(p)\wedge {\zeta }_{\kappa }(q)\}\} \\ & ={\vee }_{lm=uv}\left\{{\zeta }_{\kappa }\left(u\right)\wedge {\zeta }_{\kappa }\left(v\right)\right\} \\ & \ge {\zeta }_{\kappa }\left(l\right)\wedge {\zeta }_{\kappa }\left(m\right) \end{aligned}$$

Implies, $$\zeta \left(lm\right)\ge \zeta \left(l\right)\wedge \zeta \left(m\right).$$□

#### Proposition 4

*Let *$\zeta =({\zeta }_{1},{\zeta }_{2}, \ldots ,{\zeta }_{m})$* be an m-PF subset of *$W$*. Then *$\zeta$* is an m-PFQI of *$W$* if and only if *${\zeta }_{t} =\left\{l\in W|\zeta (W)\ge t\right\}\ne \varphi$* is a QI of *$W$* for all *$t=({t}_{1},{t}_{2},...,{t}_{m})\in (\mathrm{0,1}{]}^{m}$.

#### Proof

Let $\zeta$ is an *m*-PFQI of $W$. It is enough to prove ${\zeta }_{t}W\cap W{\zeta }_{t}\subseteq {\zeta }_{t}.$ Let $n\in {\zeta }_{t}W\cap W{\zeta }_{t}.$ Then $n\in {\zeta }_{t}W$ and $n\in W{\zeta }_{t}.$ Also $n=al$ and $n=mb$ for some $l,m\in W$ and $a,b\in {\zeta }_{t}.$ Thus ${\zeta }_{\kappa }(a)\ge {t}_{\kappa }$ and ${\zeta }_{\kappa }(b)\ge {t}_{\kappa }$ for all $\kappa \in \{\mathrm{1,2}, \ldots ,m\}.$ Now,

$$\begin{aligned}({\zeta }_{\kappa }\circ {\delta }_{\kappa })(n) &={\vee }_{n=gh}\{{\zeta }_{\kappa }(g)\wedge {\delta }_{\kappa }(h)\}\\ & \ge {\zeta }_{\kappa }(a)\wedge {\delta }_{\kappa }(l)\text{ because }n=al \\ & ={\zeta }_{\kappa }\left(a\right)\wedge 1 \\ & ={\zeta }_{\kappa }(a)\\ & \ge {t}_{\kappa }. \end{aligned}$$

So, $({\zeta }_{\kappa }\circ {\delta }_{\kappa })(n)\ge {t}_{\kappa }$ for all $\kappa \in \{\mathrm{1,2}, \ldots ,m\}$. Now,

$$\begin{aligned} ({\delta }_{\kappa }\circ {\zeta }_{\kappa })(n) & ={\vee }_{n=gh}\{{\delta }_{\kappa }(g)\wedge {\zeta }_{\kappa }(h)\} \\ & \ge {\delta }_{\kappa }(m)\wedge {\zeta }_{\kappa }(b)\text{ because }n=mb \\ &=1\wedge {\zeta }_{\kappa }(b) \\ & ={\zeta }_{\kappa }(b) \\ & \ge {t}_{k}. \end{aligned}$$

So, $({\delta }_{\kappa }\circ {\zeta }_{\kappa })(n)\ge {t}_{\kappa }$ for all $\kappa \in \{\mathrm{1,2}, \ldots ,m\}.$

Thus, $(({\zeta }_{\kappa }\circ {\delta }_{\kappa })\wedge ({\delta }_{\kappa }\circ {\zeta }_{\kappa }))(n)=({\zeta }_{\kappa }\circ {\delta }_{\kappa })(n)\wedge ({\delta }_{\kappa }\circ {\zeta }_{\kappa })(n)\ge {t}_{\kappa }\wedge {t}_{\kappa }={t}_{\kappa }$ for all $\kappa \in \{\mathrm{1,2}, \ldots ,m\}.$ So $\zeta (n)\ge \left(\left(\zeta \circ \delta \right)\wedge \left(\delta \circ \zeta \right)\right)\left(n\right)\ge t$ implies $n\in {\zeta }_{t}.$ Hence ${\zeta }_{t}$ is QI of $W.$

Conversely, on contrary suppose $\zeta$ is not *m*-PFQI of $W.$ Suppose for $l\in W, (({\zeta }_{\kappa }\circ {\delta }_{\kappa })\wedge ({\delta }_{\kappa }\circ {\zeta }_{\kappa }))(l)>{\zeta }_{\kappa }(l)$ for some $\kappa \in \{\mathrm{1,2}, \ldots ,m\}.$ Choose ${t}_{\kappa }\in (\mathrm{0,1}{]}^{m}$ such that ${t}_{k}=({\zeta }_{\kappa }\circ {\delta }_{\kappa })(l)\wedge ({\delta }_{\kappa }\circ {\zeta }_{\kappa })(l)$. This implies that $l\in {{(\zeta }_{\kappa }\circ {\delta }_{\kappa })}_{{t}_{\kappa }}$ and $l\in {({\delta }_{\kappa }\circ {\zeta }_{\kappa })}_{{t}_{\kappa }}$ but $l\notin {{(\zeta }_{\kappa })}_{{t}_{\kappa }}$ which is a contradiction. So,$(\zeta \circ \delta )\wedge (\delta \circ \zeta )\le \zeta .$

Hence, it is shown that if an m-PF subset of $W$ is m-PFQI then its level set is QI and vice versa.□

#### Lemma 10

*Every m-PF one-sided ideal of* $W$ *is an m-PFQI of* $W.$

#### Proof

The Proof Follows from Lemma 3.□

In Example 3, it is shown that the converse of Lemma 10 may not be true.

#### Example 3

Consider the semiring ${\text{W}}=\{0,a,1\}$ under the operations as given in Tables 7 and 8.

**Table 7:** Table of addition.

$+$	$0$	$a$	$1$
$0$	$0$	$a$	$1$
$a$	$a$	$a$	$a$
$1$	$1$	$a$	$1$

**Table 8:** Table of multiplication.

$*$	$0$	$a$	$1$
$0$	$0$	$0$	$0$
$a$	$0$	$a$	$a$
$1$	$0$	$a$	$1$

We define a 3-PF subset $\zeta =({\zeta }_{1},{\zeta }_{2},{\zeta }_{3})$ of ${\text{W}}$ as follows:

$$\zeta (0)=(\mathrm{0.5,0.5,0.6}),\zeta (a)=(\mathrm{0,0},0),\zeta (1)=(\mathrm{0.1,0.3,0.4}).$$

Then simple calculations show that ${\upzeta }_{{\text{t}}}$ is a QI of $W.$ Therefore by using Proposition 3$\upzeta$ is 3-PFQI of $W.$ Now, $\zeta \left(a\right)=\zeta \left(1.a\right)=\left(\mathrm{0,0},0\right)$  $\left(\mathrm{0.1,0.3,0.4}\right).=\zeta (1)$. So $\zeta$ is not a 3-PFRI of $W.$

#### Lemma 11

*Let* $\zeta =({\zeta }_{1},{\zeta }_{2}, \ldots ,{\zeta }_{m})$ *and* $\xi =({\xi }_{1},{\xi }_{2}, \ldots ,{\xi }_{m})$ *be m-PFRI and m-PFLI of* $W$ *respectively. Then* $\zeta \wedge \xi$ *is an m-PFQI of* $W.$

#### Proof

Let $\zeta =({\zeta }_{1},{\zeta }_{2}, \ldots ,{\zeta }_{m})$ and $\xi =({\xi }_{1},{\xi }_{2}, \ldots ,{\xi }_{m})$ be an *m*-PFRI and *m*-PFLI of $W$ resp. We have to show that $\zeta \wedge \xi$ is *m*-PFQI of $W.$

Now let $n\in W.$ If $n\ne uv$ for some $u,v\in W.$ Then there is nothing to prove. If $n=ef$ for some $e,f\in W.$ Then

$$\begin{aligned}((({\zeta }_{\kappa }\wedge {\xi }_{\kappa })\circ {\delta }_{\kappa })\wedge ({\delta }_{\kappa }\circ ({\zeta }_{\kappa }\wedge {\xi }_{\kappa })))(n)& =(({\zeta }_{\kappa }\wedge {\xi }_{\kappa })\circ {\delta }_{\kappa })(n)\wedge ({\delta }_{\kappa }\circ \left({\zeta }_{\kappa }\wedge {\xi }_{\kappa }\right))(n)\\ & ={\vee }_{n=ef}\{({\zeta }_{\kappa }\wedge {\xi }_{\kappa })(e)\wedge ({\delta }_{\kappa })(f)\} \\ & \wedge {\vee }_{n=ef}\{{\delta }_{\kappa }(e)\wedge ({\zeta }_{\kappa }\wedge {\xi }_{\kappa })(f)\} \\ & ={\vee }_{n=ef}\{({\zeta }_{\kappa }\wedge {\xi }_{\kappa })(e)\wedge ({\zeta }_{\kappa }\wedge {\xi }_{\kappa })(f)\} \\ & ={\vee }_{n=ef}\{(({\zeta }_{\kappa }(e)\wedge ({\xi }_{\kappa })(f))\wedge ({\zeta }_{\kappa }(f)\wedge { \xi }_{\kappa }(e))\} \\ & \le {\vee }_{n=ef}\{({\zeta }_{\kappa }(e)\wedge {\xi }_{\kappa }(f)\} \\ & \le {\vee }_{n=ef}\{({\zeta }_{\kappa }(ef)\wedge {\xi }_{\kappa }(ef)\} \\ & ={\vee }_{n=ef}\{({\zeta }_{\kappa }\wedge {\xi }_{\kappa })(ef)\} \\ & =({\zeta }_{\kappa }\wedge {\xi }_{\kappa })(n)\text{ for all }\kappa \in \{\mathrm{1,2}, \ldots ,m\}. \end{aligned}$$

Hence, $((\zeta \wedge \xi )\circ \delta )\wedge (\delta \circ \left(\zeta \wedge \xi \right))\le \zeta \wedge \xi ,$ that is $\zeta \wedge \xi$ is an *m*-PFQI of $W.$□

## Characterization of regular and intra-regular semirings by *m*-polar fuzzy ideals

In this section, regular and intra-regular semirings are characterized by *m*-PFIs. Some theorems are proved regarding regular and intra-regular semirings in terms of *m*-PFIs, *m*-PFQIs and *m-*PFBIs.

### Definition 8

$W$ is said to be regular if for all $a\in W$*,* there exist an element $l\in W$ such that $a=ala.$ ^11^.

### Theorem 1

*For semiring *$W,$* the following assertions are equivalent*^30^:

1. $$W$$* is regular*;
2. $I\cap J=IJ$* for every RI *$I$* and LI *$J$* of *$W.$

### Theorem 2

*Every m-PFQI of *$W$* is an m-PFBI of *$W$.

### Proof

Suppose that $\zeta =({\zeta }_{1},{\zeta }_{2},...,{\zeta }_{m})$ is an *m*-PFQI of $W$. Let $l,m,n\in W.$ Then,

$$\begin{aligned} ({\delta }_{\kappa }\circ {\zeta }_{\kappa }))(lmn)& ={\vee }_{lmn=uv}\{({\delta }_{\kappa }(u)\wedge {\zeta }_{\kappa }(v)\}\\ & \ge {\delta }_{\kappa }(lm)\wedge {\zeta }_{\kappa }(n) \\ & ={\zeta }_{\kappa }(n). \end{aligned}$$

So $, ({\delta }_{\kappa }\circ {\zeta }_{\kappa })(lmn)\ge {\zeta }_{\kappa }(n)$ for all $\kappa \in \{\mathrm{1,2}, \ldots ,m\}.$

Since $lmn=l(mn)\in lW=lW,$ so $(lm)n=lf$ for some $f\in W.$ Thus

$$\begin{aligned}({\zeta }_{\kappa }\circ {\delta }_{\kappa })((lmn)& ={\vee }_{lmn=uv}\{({\zeta }_{\kappa }(u)\wedge {\delta }_{\kappa })(v)\} \\ & \ge {\zeta }_{\kappa }\left(l\right)\wedge {\delta }_{\kappa }\left(f\right) \text{since }\left(lm\right)n=l \\ & ={\zeta }_{\kappa }(l)\wedge 1 \\ & ={\zeta }_{\kappa }(l) \end{aligned}.$$

So,$({\zeta }_{\kappa }\circ {\delta }_{\kappa })(lmn)\ge {\zeta }_{\kappa }(l)$ for all $\kappa \in \left\{\mathrm{1,2},...,m\right\}.$ Now, by our supposition

$$\begin{aligned} {\zeta }_{\kappa }((lmn) & \ge (({\zeta }_{\kappa }\circ {\delta }_{\kappa })\wedge ({\delta }_{\kappa }\circ {\zeta }_{\kappa }))(lmn) \\ & =({\zeta }_{\kappa }\circ {\delta }_{\kappa })(lmn)\wedge ({\delta }_{\kappa }\circ {\zeta }_{\kappa })(lmn) \\ & \ge {\zeta }_{\kappa }(l)\wedge {\zeta }_{\kappa }(n)\text{ for all }\kappa \in \{\mathrm{1,2}, \ldots ,m\}. \end{aligned}$$

Thus, $\zeta (lmn) \ge \zeta (l)\wedge \zeta (n).$ Hence $\zeta$ is an *m*-PFBI of $W.$□

### Theorem 3

*For *$W$* the following conditions are equivalent*.

1. $$W$$* is regular*;
2. $(\zeta \wedge \xi )=\zeta \circ \xi$* for every m-PFRI *$\zeta$* and m-PFLI *$\xi$* of *$W$*.*

### Proof

$1)\Rightarrow (2):$Let $\zeta =({\zeta }_{1},{\zeta }_{2}, \ldots ,{\zeta }_{m})$ and $\xi =({\xi }_{1},{\xi }_{2}, \ldots ,{\xi }_{m})$ be two *m-*PFRI and *m*-PFLI of $W$ respectively. Let $l\in W,$ we have.

$$\begin{aligned} ({\zeta }_{\kappa }\circ {\xi }_{\kappa })(l) & ={\vee }_{l=ef}\{({\zeta }_{\kappa }(e)\wedge {\xi }_{\kappa }(f)\} \\ & \le {\vee }_{l=ef}\{({\zeta }_{\kappa }(ef)\wedge {\xi }_{\kappa }(ef)\} \\ & ={\zeta }_{\kappa }(l)\wedge {\xi }_{\kappa }(l) \\ & =({\zeta }_{\kappa }\wedge {\xi }_{\kappa })(l) \mathrm{for all }\kappa \in \{\mathrm{1,2}, \ldots ,m\}.\end{aligned}$$

So, $(\zeta \circ \xi )\le (\zeta \wedge \xi ).$ Since $W$ is regular, so for each $a\in W$ there exists an element $l\in W$ such that $a=ala.$

$$\begin{aligned} ({\zeta }_{\kappa }\wedge {\xi }_{\kappa })(a) & ={\zeta }_{\kappa }(a)\wedge {\xi }_{\kappa }(a) \\ & \le {\zeta }_{\kappa }(al)\wedge {\xi }_{\kappa }(a) \mathrm{since }\zeta \mathrm{ is an }m-\mathrm{PFRI of }W \\& \le {\vee }_{a=ef}\{({\zeta }_{\kappa }(e)\wedge {\xi }_{\kappa }(f)\} \\ & =({\zeta }_{\kappa }\circ {\xi }_{\kappa })(a)\mathrm{ for all }\kappa \in \{\mathrm{1,2}, \ldots ,m\}. \end{aligned}$$

So, $$(\zeta \wedge \xi )\le (\zeta \circ \xi ).$$

$(2)\Rightarrow (1)$: Let $T$ be a RI and $U$ be a LI of $W$. Then by Lemma 2, ${C}_{T}$ is *m*-PFRI and ${C}_{U}$ is m-PFRI of $W$. Hence by hypothesis ${C}_{T}\wedge {C}_{U}={C}_{T}\circ {C}_{U}$. By Lemma 1, then ${C}_{T\cap U}= {C}_{TU}$. This implies $T\cap U=TU$. Hence by Theorem 1, $W$ is a regular semiring.□

### Theorem 4

*The following assertions are equivalent for *$W$.

1. $$W$$* is regular;*
2. $\zeta =\zeta \circ \delta \circ \zeta$* for every m-PFGBI *$\zeta$* of *$W;$
3. $\zeta =\zeta \circ \delta \circ \zeta$* for every m-PFBI *$\zeta$* of *$W.$

### Proof

$\left(1\right)\Rightarrow \left(2\right):$Let $\zeta =({\zeta }_{1},{\zeta }_{2}, \ldots ,{\zeta }_{W})$ be an *m*-PFGBI of $W$ and $a\in W.$ Since $W$ is regular, so there exists an element $l$ of $W$ such that $a=ala.$ So we have.

$$\begin{aligned} ({\zeta }_{\kappa }\circ {\delta }_{\kappa }\circ {\zeta }_{\kappa })(a) & ={\vee }_{a=uv}\{({\zeta }_{\kappa }\circ {\delta }_{\kappa })(u)\wedge {\zeta }_{\kappa }(v)\} \mathrm{for some }u,v\in W \\ & \ge ({\zeta }_{\kappa }\circ {\delta }_{\kappa })(al)\wedge {\zeta }_{\kappa }(a) \mathrm{since }a=ala \\ & ={\vee }_{al=pq}\{{\zeta }_{\kappa }(p)\wedge {\delta }_{\kappa })(q)\}\wedge {\zeta }_{\kappa }(a) \\ & \ge \{{\zeta }_{\kappa }(a)\wedge {\delta }_{\kappa }(l)\}\wedge {\zeta }_{\kappa }(a) \\ & ={\zeta }_{\kappa }(a) \mathrm{for all }\kappa \in \{\mathrm{1,2}, \ldots ,m\}. \end{aligned}$$

Hence $$\zeta \circ \delta \circ \zeta \ge \zeta .$$

Since $\zeta$ is an *m*-PFGBI of $W$. So we have $(\zeta \circ \delta )\circ \zeta \le \zeta .$ Thus $\zeta =(\zeta \circ \delta )\circ \zeta .$

$$(2)\Rightarrow (3):$$ Straightforward.

$(3)\Rightarrow (1):$ Let $\zeta , \xi$ be *m*-PFRI and *m*-PFLI of $W$, respectively. Then $\zeta \wedge \xi$ is *m*-PFQI of $W$ and every *m*-PFQI is *m*-PFBI of $W.$ So by hypothesis

$$\begin{aligned} {\zeta }_{\kappa }\wedge {\xi }_{\kappa }& =({\zeta }_{\kappa }\wedge {\xi }_{\kappa })\circ {\delta }_{\kappa }\circ ({\zeta }_{\kappa }\wedge {\xi }_{\kappa }) \\ & \le {\zeta }_{\kappa }\circ {\delta }_{\kappa }\circ {\xi }_{\kappa } \\ & \le {\zeta }_{\kappa }\circ {\xi }_{\kappa }. \end{aligned}$$

But ${\zeta }_{\kappa }\circ {\xi }_{\kappa }\le {\zeta }_{\kappa }\wedge {\xi }_{\kappa }$ always hold. Hence ${\zeta }_{\kappa }\circ {\xi }_{\kappa }={\zeta }_{\kappa }\wedge {\xi }_{\kappa }$ that is $\zeta \circ \xi =\zeta \wedge \xi .$ Thus by Theorem 3, $W$ is a regular semiring.□

### Theorem 5

*The following assertions are equivalent for *$W$.

1. $$W$$* is regular*;
2. $\zeta \wedge \xi \wedge I\le \zeta \circ \xi \circ I$* for every m-PFRI *$\zeta ,$* m-PFGBI *$\xi$* and every m-PFLI *$I$* of *$W$;
3. $\zeta \wedge \xi \wedge I\le \zeta \circ \xi \circ I$* for every m-PFRI *$\zeta ,$* m-PFBI *$\xi$* and every m-PFLI *$I$* of *$W$;
4. $\zeta \wedge \xi \wedge I\le \zeta \circ \xi \circ I$* for every m-PFLI *$\zeta ,$* m-PFQI *$\xi$* and every m-PFLI I of *$W$.

### Proof

$(1)\Rightarrow (2)$Let $\zeta =\left({\zeta }_{1},{\zeta }_{2}, \ldots ,{\zeta }_{m}\right), \xi =({\xi }_{1},{\xi }_{2},...,{\xi }_{m})$ and $I =({I}_{1}{,I}_{2}, \ldots, {I}_{m})$ be *m*-PFRI, *m*-PFGBI and *m*-PFLI of $W$ respectively. Let $a$ be an element of $W$ such that $a=ala.$ Hence we have.

$$\begin{aligned}\left({\zeta }_{\kappa }\circ {\xi }_{\kappa }\circ {I}_{\kappa }\right)\left(a\right) &={\vee }_{a=uv}\{({\zeta }_{\kappa }\circ {\xi }_{\kappa })(u)\wedge {I}_{\kappa }(v) \\ & \ge ({\zeta }_{\kappa }\circ {\xi }_{\kappa })(a)\wedge {I}_{\kappa }(la)\mathrm{ since }a=ala \\ & \ge {\vee }_{a=ef}\{{\zeta }_{\kappa }(e)\wedge {\xi }_{\kappa })(f)\}\wedge {I}_{\kappa }(la) \\ & \ge {\zeta }_{\kappa }(al)\wedge \xi (a)\wedge {I}_{\kappa }(la) \mathrm{since }a=ala \\ & \ge {\zeta }_{\kappa }(a)\wedge \xi (a)\wedge {I}_{\kappa }(a) \\ & \ge \left({\zeta }_{\kappa }\wedge {\xi }_{\kappa }\wedge {I}_{\kappa }\right)\left(a\right) \mathrm{for all }\kappa \in \{\mathrm{1,2}, \ldots ,m\} \end{aligned}.$$

Thus $$\zeta \wedge \xi \wedge I \le \zeta \circ \xi \circ I .$$

$$(2)\Rightarrow (3)\Rightarrow (4):$$ Straightforward.

$(4)\Rightarrow (1):$ Let $\zeta =({\zeta }_{1},{\zeta }_{2}, \ldots ,{\zeta }_{m})$ and $I =({I}_{1},{I}_{2}, \ldots ,{I}_{m})$ be an *m*-PFRI and *m*-PFLI of $W$ respectively. As $\delta$ is an *m*-PFQI of $W$, by the supposition, we have

$$\begin{aligned} ({\zeta }_{\kappa }\wedge {I}_{\kappa })(l) &=({\zeta }_{\kappa }\wedge {\delta }_{\kappa }\wedge {I}_{\kappa })(l) \\ &\le ({\zeta }_{\kappa }\circ {\delta }_{\kappa }\circ {I}_{\kappa })(l) \\ & ={\vee }_{l=ef}({\zeta }_{\kappa }\circ {\delta }_{\kappa })(e)\wedge {I}_{\kappa }(f) \\ & ={\vee }_{l=ef}\{{\vee }_{e=uv}{\zeta }_{\kappa }(u)\wedge {\delta }_{\kappa }(v)\}\wedge {I}_{\kappa }(f)\} \\ &={\vee }_{l=ef}\{{\vee }_{e=uv}{\zeta }_{\kappa }(u)\wedge 1\}\wedge {I}_{\kappa }(f)\} \\ & \le {\vee }_{l=ef}\{{\vee }_{e=uv}{\zeta }_{\kappa }(uv)\}\wedge {I}_{\kappa }(f)\} \\ & ={\vee }_{l=ef}{\zeta }_{\kappa }(e)\wedge {I}_{\kappa }(f) \\ & =\left({\zeta }_{\kappa }\circ {I}_{\kappa }\right)\left(l\right)\mathrm{ for all }\kappa \in \{\mathrm{1,2}, \ldots ,m\}. \end{aligned}$$

Thus $(\zeta \wedge I)\le$ $(\zeta \circ I)$ for every *m*-PFRI $\zeta$ and every *m*-PFLI $I$ of $W.$ But $(\zeta \circ I)\le (\zeta \wedge I)$ always. So, $(\zeta \circ I)=(\zeta \wedge I).$ Hence from Theorem 3, $W$ is regular.□

### Definition 9

$W$ is stated as an intra-regular semiring if for each $l\in W$ we get $l\in W{l}^{2}W$ that is $l$ can be written as $l= {\sum }_{i=1}^{n}{a}_{i}{l}^{2}{b}_{i}$ for some ${a}_{i},{b}_{i}\in W$^10^.

### Theorem 6

$W$* is intra-regular if and only if *$I\cap J\subseteq IJ$* for all RI *$I$* and LI *$J$* of *$W$^35^.

### Theorem 7

*The following assertions are equivalent for* $W$ *.*

1. $W$* is intra-regular*;
2. $\zeta \wedge \xi \le \zeta \circ \xi$ *for every m-PFLI* $\zeta$ *and m-PFRI* $\xi$ *of* $W$ *.*

### Proof

$(1)\Rightarrow (2)$ Let $W$ be an intra-regular semiring and $\zeta ,\xi$ be any *m*-PFLI and *m*-PFRI of $W$ respectively. For $l\in W$ there exist ${a}_{i},{b}_{i}\in W$ such that $l={\sum }_{i=1}^{n}{a}_{i}{l}^{2}{b}_{i}$ for some ${a}_{i},{b}_{i}\in W.$ Hence we have $({\zeta }_{\kappa }\circ {\xi }_{\kappa })(l)={\vee }_{l=ef}\{({\zeta }_{\kappa }(e)\wedge {\xi }_{\kappa }(f)\}\ge {\wedge }_{i=1}^{n}\left[({\zeta }_{\kappa }({a}_{i}l)\wedge {\xi }_{\kappa }(l{b}_{i})\right]\ge \left[({\zeta }_{\kappa }(l)\wedge {\xi }_{\kappa }(l)\right]$ $\mathrm{for all }\kappa \in \{\mathrm{1,2},...,m\}.$

Thus $${\zeta }_{\kappa }\circ {\xi }_{\kappa }\ge {\zeta }_{\kappa }\wedge {\xi }_{\kappa }.$$

$(2)\Rightarrow (1)$ Let $T,U$ be LI and RI *of *$W$*,* respectively. Then ${C}_{T}$ and ${C}_{U}$ are *m*-PFLI and *m*-PFRI of $W$*,* respectively. Now, by the hypothesis*,* ${C}_{T\cap U}={C}_{T}\wedge {C}_{U}\le {C}_{T}\circ {C}_{U}={C}_{TU}.$ Thus $T\cap U\subseteq TU$. So by Theorem 2, $W$ is intra-regular.□

### Theorem 8

*The following assertions are equivalent for *$W$^35^.

1. $W$* is both regular and intra-regular*;
2. $T=TT$* for each BI *$T$* of *$W.$

### Theorem 9

*The following assertions are equivalent for* $W$ *.*

1. $W$* is both regular and intra-regular*;
2. $\zeta \circ \zeta =\zeta$ *for each m-PFBI* $\zeta$ *of* $W$ *.*

### Proof

$\left(1\right)\Rightarrow \left(2\right)$ Let $W$ be both regular and intra-regular semiring and $\zeta$ be *m-*PFBI of $W$*.* For $u\in W$ there exist elements $l,{a}_{i},{b}_{i}\in W$ such that $u=ulu$ and $u={\sum }_{i=1}^{n}{a}_{i}{u}^{2}{b}_{i}.$ Thus $u=ulu=ululu=ul({\sum }_{i=1}^{n}{a}_{i}{u}^{2}{b}_{i})lu=(ul{a}_{i}u)(u{b}_{i}lu).$

Hence, we have

$$\begin{aligned}\left({\zeta }_{\kappa }\circ {\xi }_{\kappa }\right)\left(u\right) & ={\vee }_{u=ef}\{({\zeta }_{\kappa }(e)\wedge {\xi }_{\kappa }(f)\} \\ & \ge {\wedge }_{i=1}^{n}\left[({\zeta }_{\kappa }(ul{a}_{i}u)\wedge {\zeta }_{\kappa }(u{b}_{i}lu)\right]\} \\ & \ge {\wedge }_{i=1}^{n}\{({\zeta }_{\kappa }(u)\wedge {\zeta }_{\kappa }(u)\} \\ & ={\zeta }_{\kappa }\left(u\right)\mathrm{ for all }\kappa \in \{\mathrm{1,2},...,m\}. \end{aligned}$$

This implies that ${\zeta }_{\kappa }\le {\zeta }_{\kappa }\circ {\zeta }_{\kappa }$*.* But, ${\zeta }_{\kappa }\circ {\zeta }_{\kappa }\le {\zeta }_{\kappa }$ always holds. Thus ${\zeta }_{\kappa }\circ {\zeta }_{\kappa }={\zeta }_{\kappa }.$

$(2)\Rightarrow (1)$ Let $B$ be any BI of $W$*.* Then ${C}_{B}$ is *m-*PFBI of $W$*.* By the assumption ${C}_{B}={C}_{B}\circ {C}_{B}={C}_{{B}^{2}}$ Thus $B={B}^{2}$*.* Hence by Theorem 8, $W$ is both regular and intra-regular.□

## Comparative study

In this section we provide the comparative study between our newly generated results and the other results addressed by Bashir et al.^31,32^. While comparing we would like to emphasize on couple of points. An *m*-PF set in semirings is presented to overcome the restrictions involved in single-valued and two-valued fuzzification find the ideals of semigroup in terms of m-polar fuzzy ideals. However, we introduce the concept of *m*-polar fuzzy ideals in the structure of semirings. No doubt our provided results are efficient and more generalized because it tackles more complicated problems. Our methodology offers a broad variety of applications.

## Conclusion

An *m*-PF (multi-polar fuzzy) set theory is a powerful mathematical tool for decision-making and handling uncertainty in real-world scenarios where data stems from m-factors (m > 2). Bashir et al. introduced m-polar fuzzy ideals of semigroup and multi-polar fuzzy ideals of ternary semigroup^31,32^. By extending the algebraic structure, we introduced m-polar fuzzy ideals of semiring with two binary operations “addition” and “multiplication”. Within this framework, we have established significant findings relating to *m*-PF subsemirings, ideals, generalized bi-ideals, bi-ideals, and quasi-ideals within the context of semirings. Additionally, we have explored the characterizations of regular and intra-regular semirings in terms of *m*-PFIs.

In future, we will extend our research to *m*-polar Intuitionistic fuzzy set, *m*-polar Pythagorean fuzzy set, *m*-polar Picture fuzzy set and *m*-polar Spherical fuzzy set of many algebraic structures. An *m*-PF set has only membership degree for any situation but in real life there are many problems which are handled using non-membership degree. For this reason, we will use more advanced techniques in future. Further, we will explore the m-PF ideals in hyperstructures, and investigate the concept of roughness as it applies to m-PF ideals of semirings. These foundations not only strengthen the mathematical framework but also cover the way for practical applications in various fields.

## Data Availability

The datasets used and analyzed during the study available from the corresponding author on reasonable request.
